# Quantitatively Plotting the Human Face for Multivariate Data Visualisation Illustrated by Health Assessments Using Laboratory Parameters

**DOI:** 10.1155/2013/390212

**Published:** 2013-12-18

**Authors:** Wang Hongwei, Liu Hui

**Affiliations:** ^1^Department of Computer Science, Dalian Medical University, Dalian 116044, China; ^2^College of Medical Laboratory, Dalian Medical University, Dalian 116044, China

## Abstract

*Objective*. The purpose of this study was to describe a new data visualisation system by plotting the human face to observe the comprehensive effects of multivariate data. *Methods*. The Graphics Device Interface (GDI+) in the Visual Studio.NET development platform was used to write a program that enables facial image parameters to be recorded, such as cropping and rotation, and can generate a new facial image according to *Z* values from sets of normal data (*Z* > 3 was still counted as 3). The measured clinical laboratory parameters related to health status were obtained from senile people, glaucoma patients, and fatty liver patients to illustrate the facial data visualisation system. *Results*. When the eyes, nose, and mouth were rotated around their own axes at the same angle, the deformation effects were similar. The deformation effects for any abnormality of the eyes, nose, or mouth should be slightly higher than those for simultaneous abnormalities. The facial changes in the populations with different health statuses were significant compared with a control population. *Conclusions*. The comprehensive effects of multivariate may not equal the sum of each variable. The 3*Z* facial data visualisation system can effectively distinguish people with poor health status from healthy people.

## 1. Introduction

Data visualisation is the study of visual representation of data to communicate information clearly and effectively through graphical means [1–3]. The medical sciences have a uniquely intertwined relationship with bioinformatics. The rapidly expanding field of biology creates enormous challenges to enable researchers to gain insights from large and highly complex data sets. Although researchers and practitioners often create patterns that can be visually identified, such as charts, graphs, and interactive displays, when solving a large range of problems, there are no definite accepted methods to identify these complex relationships [4–6].

Traditional data visualisation systems are mostly based on mathematical models, but complex bioinformatic correlations may not follow previously known statistical rules. Therefore, it is essential to explore methods for data visualisation that do not completely rely on mathematical models. We established the 3*Z* facial data visualisation system based on changes in human facial features. Certain specific bioinformatics rules for correlations may be elucidated with the use of the data visualisation system.

Chernoff first developed the idea of using human facial characteristics as a means to visualise data [7, 8]. The idea behind using faces is that humans easily recognise faces and notice small changes without difficulty. Chernoff faces display multivariate data in the shape of a human face. However, the effectiveness of this form of visualisation is still open to speculation. When assigning several variables to various facial features when drawing Chernoff faces, different drawers may make different choices. Accordingly, different grouping results may be obtained. Therefore, Chernoff faces may not work well to detect the comprehensive effects of multivariate data, and updates of facial data visualisation are required for this purpose.

Displaying data quantitatively is required for data visualisation systems. Thus, it is important to quantitatively plot the human face based on multivariate data. The correct relations of multivariate data should not be obtained without quantitative data display. We developed a program that enables facial image parameters to be recorded quantitatively according to *Z* values from sets of normal data and can generate a new facial image for observing comprehensive effects of multivariate data. Thus, a new analytic platform may elucidate complex bioinformatic correlations.

From a public point of view, the health status of an individual is influenced by social, psychological, and biological factors [9–11]. Thus, any quantitative evaluation of health status is a challenging task. Deteriorations in health status for any reason will induce changes in somatic biological factors, with a consequently increased risk of disease. This process is accompanied with changes in clinical laboratory parameters [12–14]. The present study was performed to elucidate the parameters of a facial data visualisation system based on changes in the facial features of individuals and their role in health assessment by assessing correlations with laboratory parameters.

## 2. Methods

### 2.1. Construction of the Facial Data Visualisation System

The data visualisation system was constructed on the basis of human facial images, including the eyes, nose, and mouth. These three features were rotated clockwise around their own axes, with the original value of 0° and the terminating value of 45°. Data from different groups were normalised as 0–45, and the larger the value, the larger the angle of rotation and the more significant the facial changes. The present study randomly inputted the three groups of data from both eyes, the nose, and the mouth to form the data visualisation system.

We used object-oriented technology to develop a graphics processing program with the technical support of the Microsoft.NET Framework and Graphics Device Interface (GDI+) of the Visual Studio.NET development platform. The program enabled us to develop parameters that could process graphics or images, such as cropping, rotation, and the generation of a new image, to achieve data visualisation.

### 2.2. Laboratory Parameters for Health Assessment

The related experimental indicators of organ function, blood lipid levels, and stress levels were divided into three groups to evaluate the health status from three dimensions to reflect the impact of different factors on health. Evaluation indicators of organ function included albumin (Alb, 41 ± 3.5 g/L). Evaluation indicators of blood lipids included cholesterol (Chol, 5.0 ± 0.9 mmol/L), and evaluation indicators of stress included the neutrophil count (Neut, 3.5 ± 1.2× 10^9^/L). Laboratory indices were measured with an automatic analyser in the clinical laboratory of our university hospital using standard commercial reagent kits.

### 2.3. Standardisation of Measurement Values

Based on the reference ranges, we obtained the mean values and the standard deviation (SD) for the normally distributed data. *Z*-values were calculated according to the following formula for Neut and Chol, where *X* was the measured value and Mean was the mean value:

(1) $$\begin{array}{c} Z=\frac{\left(X-\text{Mean}\right)}{\text{SD}}. \end{array}$$

For Alb, a lower measured value represented a worse health status; therefore, the *Z* values were calculated according to the following formula:

(2) $$\begin{array}{c} Z=\frac{\left(\text{Mean}-X\right)}{\text{SD}}. \end{array}$$

Thus, the measured values for the parameters as mentioned were comparable after transformation, where *Z* < 0 indicated that it was normal. The larger the value is, the poorer the health status is.

### 2.4. The Angle Transformation

*Z* > 3 indicated that the health status was very poor or the patient was ill. Therefore, emphasis was placed on *Z*-score changes between 0 and 3 in assessing health status. Therefore, *Z* < 0 was counted as 0. *Z* > 3 was counted as 3. The transformed value after the multiplication of *Z* by 15 was between 0 and 45, which was suitable for the facial data visualisation system. The measured values for different observed subjects were normalised (*Z* value transformation), and the transformation was made for the angle 0–45°(*Z* × 15).

The transformed values of angles were input into the facial data visualisation system, and thus, the correlations between the values from different dimensions and the possible integrative effects were observed.

### 2.5. Subjects

Fifteen patients with glaucoma requiring ophthalmologic surgery (57.8 ± 7.2 years old, 7 male and 8 female) were randomly selected as the glaucoma group.

Forty individuals with nonalcoholic fatty liver as indicated by ultrasonography (31.4 ± 4.3 years old, 20 male and 20 female) were selected as the fatty liver group. Subjects in the fatty liver group who met the following criteria were excluded from the study: (1) those suffering from other liver diseases, such as viral hepatitis; (2) subjects who had a dependence on alcohol; and (3) individuals aged >40 years old.

Forty elderly subjects without organic disease as detected by imaging examinations (82.5 ± 2.4 years old, 20 male and 20 female) were randomly selected as the elderly group.

In the control group, there were 40 individuals (20 male and 20 female) aged between 20 and 30 years old without organic disease as detected by imaging examinations, and their average age was 26.7 years old.

### 2.6. Statistical Analysis

The null hypothesis was that means in different populations were equal. One-way ANOVA was used to analyse the differences of each indicator among the various populations. The degrees of freedom were 3 and 156, respectively, in our sample; *F*_0.05(3,150)_ was 2.66. A difference was considered to be statistically significant when the *P* value was less than 0.05. Statistical analyses were performed with SPSS statistical analysis software for Windows (SPSS, Chicago, IL, USA).

## 3. Results

**Figure 1:**
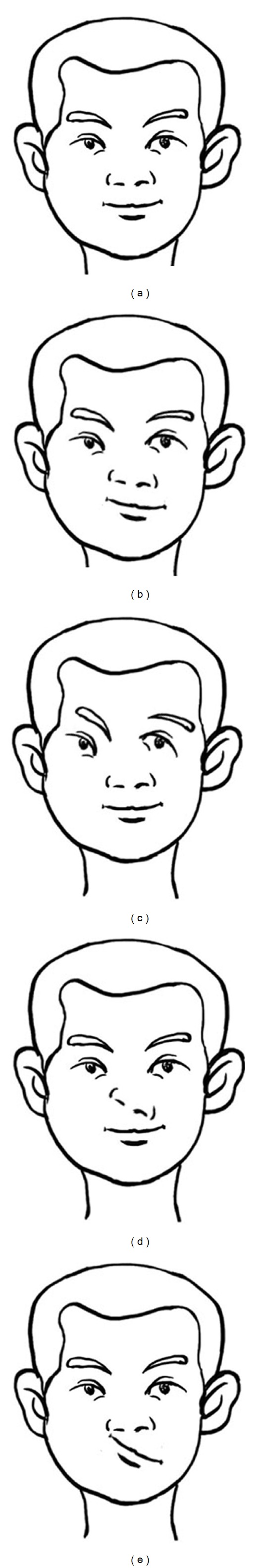
Effectiveness of the data display using the facial data visualisation system. (a) Normal value (eye = 0; nose = 0; mouth = 0); (b) facial features were rotated 10° (eye = 10; nose = 10; mouth = 10); (c) the eyes were rotated 30° (eye = 30; nose = 0; mouth = 0); (d) the nose was rotated 30° (eye = 0; nose = 30; mouth = 0); (e) the mouth was rotated 30° (eye = 0; nose = 0; mouth = 30).

The intentionally preset data (normal value; the organs were rotated 10°, the eyes were rotated 30°, the nose was rotated 30°, and the mouth was rotated 30°) were input into the facial data visualisation system. The consequent facial changes are shown in Figure 1. The deformation extent for (b) was relatively mild, whereas the deformation extents for (c)–(e) were relatively major, and the deformation extents for (c), (d), and (e) were identical.

**Figure 2:**
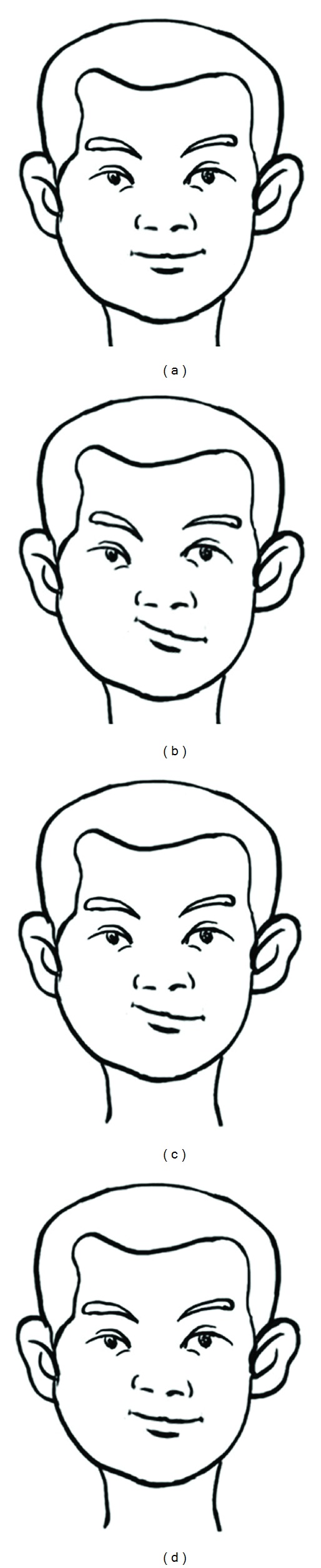
Effectiveness of the display of measured values for the populations with different health statuses using the facial data visualisation system. (a) Normal control; (b) glaucoma group; (c) fatty liver group; (d) elderly people.

The measured data for the populations with different health statuses are shown in Table 1. The measured values were converted into the angle transformation using (1), (2), and the formula described at the angle transformation section in Method. The median angle values in different groups are also shown in Table 1 and were input into the facial data visualisation system (rotation of eye depends on data of Neut; that of nose depends on data of Alb, and that of mouth depends on data of Chol). The resulting facial changes are shown in Figure 2. The facial changes in the populations with different health statuses were significant compared with those in the control populations.

## 4. Discussion

To assess the comparability of change in measured values, the data transformation into *Z* values was conducted using the average value and standard deviation. Because the maximal *Z* value preset was 3 in our system, the data visualisation system was defined as a 3*Z* facial data visualisation system. The positions of human facial features (eyes, nose, and mouth) constitute the major facial characteristics, and the main mode of recognition is in the brain. Thus, observers are sensitive to facial deformation. The present study rotated the eyes, nose, and mouth around their own respective axes, and significant deformation effects should be found when *Z* = 2 according to statistical theory. The observations (*Z* = 2) made in Figures 1(c)–1(e) [(c), eyes rotated 30°; (d), nose rotated 30°; and (e), mouth rotated 30°] revealed that the deformation effects from the rotating angles of the eyes, nose, and mouth on the face were similar, which indicated that the rotation of these features can be used to observe the comprehensive effects of multivariate data.

As seen in Figure 1, the sum of the rotations for eyes, nose, and mouth was 30° when they were rotated 10° separately (Figure 1(b)), and the deformation effects should be identical to those shown in Figures 1(c), 1(d), or 1(e). However, the results reveal that the deformation extent in Figure 1(b) was significantly lower than those shown in Figures 1(c), 1(d), or 1(e), which indicates that the deformation effects for any abnormality in the eyes, nose, or mouth should be higher than that for small simultaneous abnormalities. Therefore, the display of data with the use of facial characteristics may unravel new data patterns. Concerning the complex objects affected by multiple factors, the influence on the subject from significant changes in a certain factor may be higher than that from small changes in several factors; therefore, the facial data visualisation system may be more suitable for the analysis of certain complex systems.

Changes in health status usually do not accompany specific clinical signs. Routinely measured clinical laboratory parameters may be the only useful information for the assessment of healthy status of individuals [14–16]. However, the diagnostic thresholds usually provided are used for diagnosing diseases rather than assessing healthy status. Our results indicate that the *P* values were all less than or near 0.05 for the 3 indicators for the populations with different health statuses, indicating that these 3 indicators can be used for health assessment in distinguishing people with good and poor health. The problem was that we could not define the degree of poor health status using these 3 indicators. The health status of individuals may be determined by the quantity of the dimension with the poorest indication for health or the average of different dimensions. Therefore, the objective and accurate evaluation of health status becomes difficult.

As the experimental data in this study were represented by values, they were suitable for data visualisation. The facial data visualisation system may be more suitable for displaying and analysing health status parameters. We selected patients with glaucoma as a representative stress population, patients with fatty liver as a representative overnutrition population and elderly subjects as a representative population with functional insufficiency, because the health status of individuals is determined by multiple factors. The present study used the parameters of Neut, Chol, and Alb to represent the stress-response level, blood lipid level, and organ function level of the subjects, respectively [15–17], in three populations with different health statuses, compared with normal controls. Any item from these three indices among the populations may be different from that of normal population, although the degrees were not the same. The measured values for the populations as mentioned above were displayed by using the facial data visualisation system. The results indicated that the facial changes in the control population were not significant, whereas the facial changes in the populations with different health statuses were significant (Figure 2). The facial data visualisation system could effectively distinguish people with a poor health status from normal people, which indicates that this system may be a valid tool for the analysis of complex systems.

This facial data visualisation system will primarily be used for individual evaluation, rather than population evaluation. Therefore, the relatively small sample sizes in our study were acceptable for obtaining features of different health statuses. The limitation of our facial data visualisation system was that the system has only three rotational variables and was not suitable for displaying more parameters. We suggest selecting three factors that explain most of the variance observed in a much larger number of manifest variables with the factor analysis method first and then, using our facial data visualisation system, displaying these three factors. Although the factor analysis technique could solve the above problem, the development of a facial data visualisation system with more variables is still an important future research direction.

## 5. Conclusions

In this work, we explore the way for data visualization that does not completely rely on mathematical models. The facial data visualization system based on quantitative changes in human facial features has been established. A deeper understanding of multivariate data could be obtained by plotting facial image system through intuitive experience. Certain specific bioinformatics rules for correlations could also be unraveled with using this data visualization system. The facial data visualization system may effectively distinguish people with a poor health status.

## Figures and Tables

**Table 1:** Measured data and angular transformation for the populations with different health statuses.

Group	Measured value (mean ± SD)			Angular transformation (median)		
	Neut	Alb	Chol	Neut	Alb	Chol
Glaucoma	5.09 ± 1.86	39.11 ± 4.57	6.11 ± 1.51	11.0	4.0	18.5
Fatty liver	3.73 ± 1.35	40.29 ± 3.06	5.86 ± 1.13	0.1	1.1	12.9
Elderly	3.67 ± 1.32	38.62 ± 2.28	5.61 ± 1.09	0.0	9.5	7.9
Control	3.33 ± 1.04	40.99 ± 2.48	5.31 ± 1.39	0.0	1.8	0.5

*F* value	6.513	5.116	2.102	—	—	—
*P* value	<0.0001	0.002	0.103	—	—	—

*F*_0.05(3,150)_ = 2.66.
